# Scaling and sampling dependencies of forest canopy height mapping towards jurisdictional biomass reporting using airborne LiDAR and small-area estimation

**DOI:** 10.1186/s13021-025-00370-9

**Published:** 2025-12-08

**Authors:** Juan Guerra-Hernández, Francisco Mauro-Gutiérrez, Francisco Rodríguez-Puerta, Adrian Pascual

**Affiliations:** 1 Forest Research Centre, Associate Laboratory TERRA, School of Agriculture, University of Lisbon (ISA), Tapada da Ajuda, Lisboa, 1349-017 Portugal; 2 EiFAB, School of Forestry, Agronomy and Bioenergy Engineering, University of Valladolid, Soria, Spain; 3 Sustainable Forest Management Research Institute, iuFOR, University of Valladolid, Soria, Spain; 4 Department of Geographical Sciences, University of Maryland, College Park, MD USA

**Keywords:** Inventory plots, Forest statistics, Resolution dependence, Forest statistics, SAE, Reporting

## Abstract

Consolidated airborne laser scanning (ALS) programs, satellite imagery and spaceborne structural measurements have enabled major advances in canopy height mapping that translate towards the forest carbon biomass arena. However, we must carefully evaluate the cost of using fine-grained canopy height products to predict biomass under calibration models scoped at the scale of inventory plots. In this study, we estimated biomass using field plots and ALS metrics before predicting biomass over a jurisdiction of ~ 15,500 km^2^ in Spain using 10 m, 25 m, 44 m, and 100 m as prediction scales. We altered the scale of ALS-based biomass predictors in 10 sub-jurisdictions intensively surveyed by the Spanish National Forest Inventory (NFI) before estimating mean and total biomass using three options: (i) traditional NFI design-based (DB) estimation, (ii) a model-based (MB) approach using scale-varying canopy height metrics from ALS and NFI plots, and (iii) an small-area estimation (SAE) implemntation designed for sub-jurisdictional domains. Higher uncertainties - relative standard errors (SE) - were found for DB, particularly at sub-jurisdictional and stratum levels. We observed a consistent increase in uncertainty for MB estimation from the finest 10 m scale up to 100 m. In MB estimation, the maximum relative bias reached 11% for 10-m predictions compared to the baseline estimate at the NFI sampling native resolution. The bias associated with the prediction scale ranged from + 5% (25 m) to -8% (100 m). The mean biomass estimates for SAE generally ranged between DB and MB but at lower uncertainty to the former, especially as the NFI sampling becomes scarcer and not enough for solid inference of biomass mean. The SEA statistics helped to disentangle biomass comparisons between ALS-based inference and the traditional NFI estimation that do not incorporate remote sensing data.

## Introduction

 One of the critical aspects of terrestrial ecosystems monitoring is the estimation of above-ground biomass density (AGBD) using three-dimensional (3D) vertical vegetation structure metrics [1–4]. The large-scale mapping of AGBD and forest inventory variables usually relies on airborne laser scanning (ALS) data when available [5] or on global spaceborne metrics [6]. Canopy height metrics from ALS data collocated to field biomass plots are used to calibrate biomass models [7]. Biomass predictions are then summarized to estimate mean and total stock for jurisdictions [8, 9], which provides validation data for global biomass products and country statistics [9–11]. Methods to map canopy height from remote sensing platforms are in expansion i.e., GEDI metrics [12], fused products combining GEDI and/or spectral metrics from satellite missions and commercial applications [13–18] or ALS-powered surveys programs [19–21]. The precision and representativeness of canopy height/biomass relationships is what brings more uncertainty into biomass maps and estimates considering the complexity of biomass stocking across hierarchical planning levels and jurisdictional boundaries [22–26].

The calibration of biomass using ALS canopy height and structural statistics can be impacted by multiple factors such as the precision of tree measurements and species identification [27–29], geolocation and collocation of response and predictor variables [30–32], temporal mismatches and seasonality [33, 34] or resolution dependencies between reference data and predictors derived from remote sensing data (e.g [35–37]).,. This issue of resolution mismatch is particularly relevant when large plots from National Forest Inventories (NFIs) are the source to calibrate and validate high-resolution estimates derived from remote sensing [38, 39]. For instance, the 25-m footprint of GEDI measurements can be smaller than average sampling sizes in designed-based NFIs used to capture large variances in populations [40]. Similarly [41], upscaled a high-resolution canopy height product to 30 m before applying biomass models across Europe trained with NFI plots that did not match the proposed scale of 30 m. These and other implementations have been enabled by cutting-edge forest mapping using spaceborne and airborne LiDAR products [42–44] and are contingent to height/biomass calibrations conducted at NFI sampling scales.

A key enabling factor for implementing ALS-supported biomass estimation is the flexibility of ALS-based forest inventory: the upscaling/downscaling of ALS height and density statistics is not a computational barrier with modern processing tools [45] as long as ALS coverage is sufficiently dense and uniform across jurisdictions [23, 46, 47]. Moreover, the data point density is improving across consolidated survey programs. For instance, the third nationwide coverage in Spain will double, at least, the point density from past ALS acquisitions [21]. While dense ALS-based products are desirable in forest mapping to account for disturbances and ecological dynamics [48], it is questionable whether biomass can be accurately predicted at scales different from the sampling size used for model training [37].

The scale dependency of ALS-based prediction models is revealed by a simple observation that the aggregation of fine-grained e.g., CHM values or a given height percentile, is not equal to the single value over the aggregated area (i.e [37, 48, 49].,. The study from Packalen et al., (2019) [46] aggregated tree-level data to test the impact of resolution of canopy height predictors in biomass estimates and predictions. The reported errors were low although the experiment was not representative of jurisdictional reporting as in [50]. We strive here for a more comprehensive evaluation of scale-invariance problems using a wider spectrum of forest types/strata and topographical gradients that can accentuate biomass uncertainties from inference to prediction [7, 51].

We framed our study in heterogeneous Mediterranean forest landscapes prone for uncertainty in biomass estimations by forest type using ALS data sources [7]. For large jurisdictions, country-level NFI grids ensure an unbiased estimation of mean and total biomass stocks. However, at lower hierarchical levels i.e., municipalities, forest strata or at management units, country-level NFI grids provide few inventory plots actionable for biomass estimation. In these cases, relying on minimal representation of samples to conduct statistical inference alone is risky and unreliable [52–54]. Small-area estimation methods (SAE) powered with ALS metrics strengthen the statistical inference of mean and total stocks using limited reference inventory plots (e.g [36, 55, 56]).,. Estimates from SAE have a validation role in the interface between design-based NFI estimation and alternative model-based estimates integrating ALS and other remote sensing data [53, 57–61].

In this study, we tested the scale-invariance of biomass estimates using an entire province in Spain - an heterogenous landscape of mixed Mediterranean and temperate forests - recently measured by the Spanish NFI and surveyed with dense ALS data. We tested the consistency of biomass predictions by altering the scale at which resolution-sensitive predictors from ALS data are computed. The explored scales ranged from 10 m to 100 m, matching the properties of global biomass and canopy height maps such as the ESA Climate Change Initiative (CCI [62]), biomass mission (100 m), Landsat and GEDI products (30 m [63]),, the commercial biomass product from Planet (30 m [41],) or the high-resolution (10 m) CHM build by [64] using satellite images and GEDI data. Jurisdictional boundaries (municipalities) and strata (forest types) were used to summarize mean and total biomass computed from the following alternatives:

i) Design-based estimation of mean and total biomass using NFI data alone;
ii) Model-based inference using biomass predictions from calibration models developed using NFI biomass and ALS statistics computed at different scales;
iii) SAE inference based on NFI and ALS metrics but scoped for lower hierarchical levels in forest management planning (strata and municipalities).

Our endeavors are to (i) disentangle comparisons of statistics used in biomass reporting, (ii) show limitations of sparse NFI sampling at producing biomass baselines for jurisdictions and (iii) test the scale-invariancy of biomass predictions using multi-resolution grids of ALS metrics designed to mimic the resolution of modern canopy height products potentially actionable for biomass mapping [51].

## Materials and methods

### Study area

**Fig. 1:**
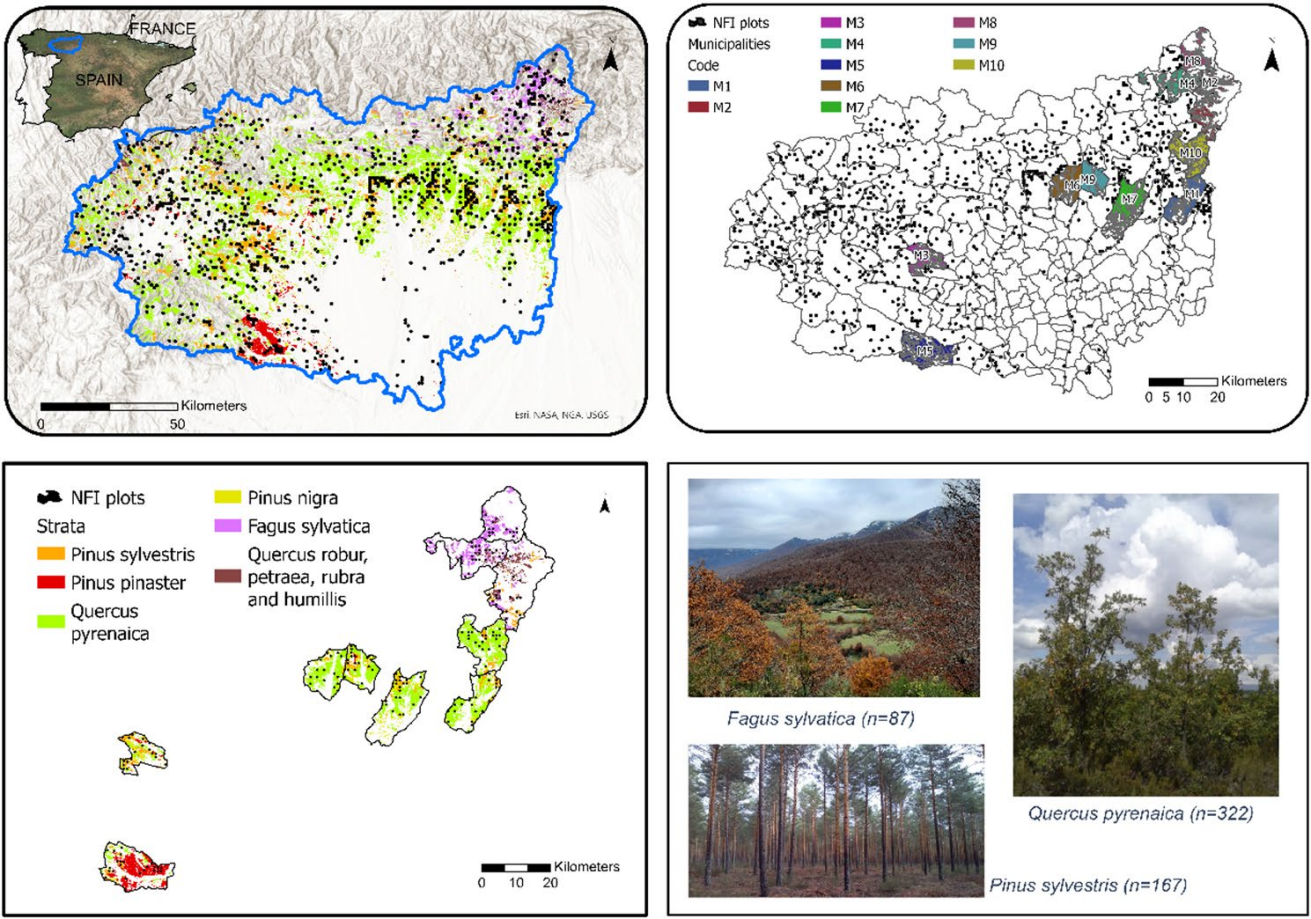
The province of Leon in NW Spain is the study region for the research. We used the NFI sampling for the six most represented forest strata and their intersection with the 10-most surveyed sub-jurisdictions (municipalities). The experiment covered from conifers and broadleaf species along and across gradients of topographical conditions, climate, historical forest management regimes, stewardship and protection

The study area is Leon jurisdictional province in the North-West of Spain (Fig. 1). Located in the Southern border of the Cantabric mountains, Leon is the seventh largest province in Spain and covers 586,000 ha of forests, mainly broadleaved species (71%) followed by coniferous (26%) and mixed forests (5%). According to Spanish Forest Map (SFM), 39 forest strata exist in the province, covering from low-land sparse oak forests to dense pine stands or shade-tolerant species (e.g., beech) in mountain forests. The slope and terrain elevation gradients cover from low-altitude agricultural plains to high mountainous ridges in the Picos de Europa National Park that exceed 2,500 m in elevation. We focused our analyses within the ten most NFI-surveyed sub-jurisdictions (municipalities) of the province (Table S1) and in the six most represented forest strata in these sub-jurisdictions (Table 1). The strata within municipalities ranged between 387.48 ha to 9,042.84 ha (2,691 ha, average) and had a sampling fraction (i.e., area of NFI plots divided by the stratum area) of 0.08% (Table S2). When combining strata across jurisdictions, we reached the average size of 11,000 ha. The study area showed larger variability than previous studies, for example [65], examined small areas ranging from 68,000 ha to 250 hectares, employing a minimum sampling fraction of 2%.

**Table 1:** Definition of main forest strata (FS) in Leon Province and sampling intensity in the latest round of the Spanish National forest inventory (NFI) for Leon Province and 10 selected jurisdictions

Forest strata (FS)	Dominant tree species	Area (ha)		NFI plots	
		Leon province	Jurisd. (10)	Leon province	Jurisd. (10)
102	*Pinus sylvestris* L. – Scots pine	64,012.82	8,794.10	167	40
103	*Pinus pinaster* – Resin pine	23,413.33	7,998.96	58	20
106	*Quercus pyrenaica* Wild.	212,017.32	32,680.84	322	66
118	*Pinus nigra subsp. Salzm.* – Corsican pine	28,631.40	7,840.99	95	26
119	*Fagus sylvatica* Wild.	21,894.53	9229.60	87	42
121	*Quercus spp: robur*,* petraea*,* rubra and humillis*	15,010.45	4629.24	47	14
FT Combined		364,979.85	71,173.73	725	208

### Biomass calibration using NFI plots and airborne lidar metrics

The calibration of biomass follows the traditional area-based approach in forest inventory making use of field plots of enhanced geolocation [31]. The Spanish National Forest Inventory (NFI) provides, among other variables, tree structural measurements for concentric plots of a maximum of 25-m radius following a constant 1-km sampling grid nationwide. A point in the 1-km grid is measured if forest cover exceeds the 10% threshold according to aerial photo interpretation. Biomass tree estimates for the plots are based on species-specific allometries. For this study, we used the latest survey collected between August-November 2019. We used 725 plots of enhanced geolocation within the 10 municipalities assessed out of the entire set of 1,160 plots available for Leon province [39]. These enhanced-geolocated plots were used to derive metrics from airborne LiDAR data on canopy height, structure and cover for the extent of the NFI plots (Table 1).

The ALS data is publicly-available through the Land Survey Institute (*Plan Nacional de Ortofotografía Aérea*, PNOA). For this study, we used the latest 2021 PNOA campaign for the province of León, acquired with the RIEGL LMS-Q1560 sensor. The dataset has an average point density of 4.7 points per square meter. This collection presents negligible temporal and/or phenological mismatches to the NFI measurements used as ground-truth for biomass calibration. Standard routines in ALS-based forest inventory [31] were used to generate the height metrics and density metrics from ALS-gridded (1 km x 1 m) and collocated to the maximum 50-m diameter of the NFI plots. The canopy height models maps were produced using pit-free algorithm implemented in lidR package at Regional level for10 m, 25 m, 44 m, and 100 m resolution [45](Figure S1),

The NFI uses a minimum threshold of 10% on forest cover measurable NFI plotlocations. Hence, we filtered our NFI plots for which ALS-based forest cover was below 10% when using 4-m height threshold to compute the height and cover statistics for biomass calibration. Biomass calibration models included two predictors to generate robust and parsimonious models. The ALS-based models were fitted using non-linear regression for dominant strata (Table 2). The leaps R package [66] was used to select variables. Collinearity between regressors was prevented by discarding candidate variables with variance inflation factor (VIF) >10. We used the absolute and relative root mean square error (RMSE) and bias as core model selection criteria. The variable selection strategy and the implementation of the area-based approach (ABA) using ALS data followed the methodology established in our previous work on LiDAR-assisted biomass estimation and uncertainty analysis in Mediterranean Forests [7, 23].

**Table 2:** Models to calibrate aboveground biomass density (AGBD, Mg ha^−1^) mesured in field plots using airborne laser scanning (ALS) statistics. Fitting results for mean squared error (RMSE, total and relative) and bias are presented for the six more dominant forest strata (FS) in Leon Province (NW of Spain)

FS code and number of field plots	Biomass density calibration models (AGBD, Mg ha^−1^) =$\:\varvec{a}\:\times\:{{\varvec{h}}_{1}}^{\varvec{b}}{{\times\:\varvec{h}}_{2}}^{\varvec{c}}$								
	Selected predictors		Parameter values			Fitting statistics			
	h_1_	h_2_	a	b	c	RMSE	RMSE (%)	Bias (%)	Adj.*R*^2^
Scots pine (102) *n* = 167	*h*_*50*_	*FC*_*ALS*_	0.0096^***^	1.0555^**^	1.5165^***^	21.07	27.50	−0.47	0.88
Resin pine (103) *n* = 58	*h*_*20*_	*FC*_*ALS*_	0.1002^***^	1.1347^**^	1.0145^***^	22.39	33.65	−0.98	0.88
Pyrenean oak (106) *n* = 325	*h*_*30*_	*FC*_*ALS*_	0.0164^***^	1.7494^**^	1.1383^***^	33.30	51.60	−2.05	0.76
Corsican pine (118)/*n* = 68	*h*_*25*_	*FC*_*ALS*_	2.3993^***^	1.1252^***^	0.3476^***^	32.00	27.42	−0.65	0.87
European beech (119)/*n* = 87	*h*_*40*_	*FC*_*ALS*_	0.0069^***^	0.5667^**^	1.9600^***^	70.90	37.30	0.84	0.84
European oak (121)/*n* = 47	*h*_*50*_		2.6310^*^	1.7440^**^		72.39	45.03	0.69	0.70

h_p_ represent the *p* height percentile distribution of ALS echoes in the plots and *FC*_*ALS*_ is the proportion of ALS echoes classified as first and ranging at least 2 m aboveground over the total number of ALS echoes classified as first ranging with each NFI plot

Significance level at *p* < 0.01, ** significance level at *p* < 0.005, *** significance level at *p* < 0.001

### Multi-resolution canopy height predictors

**Fig. 2:**
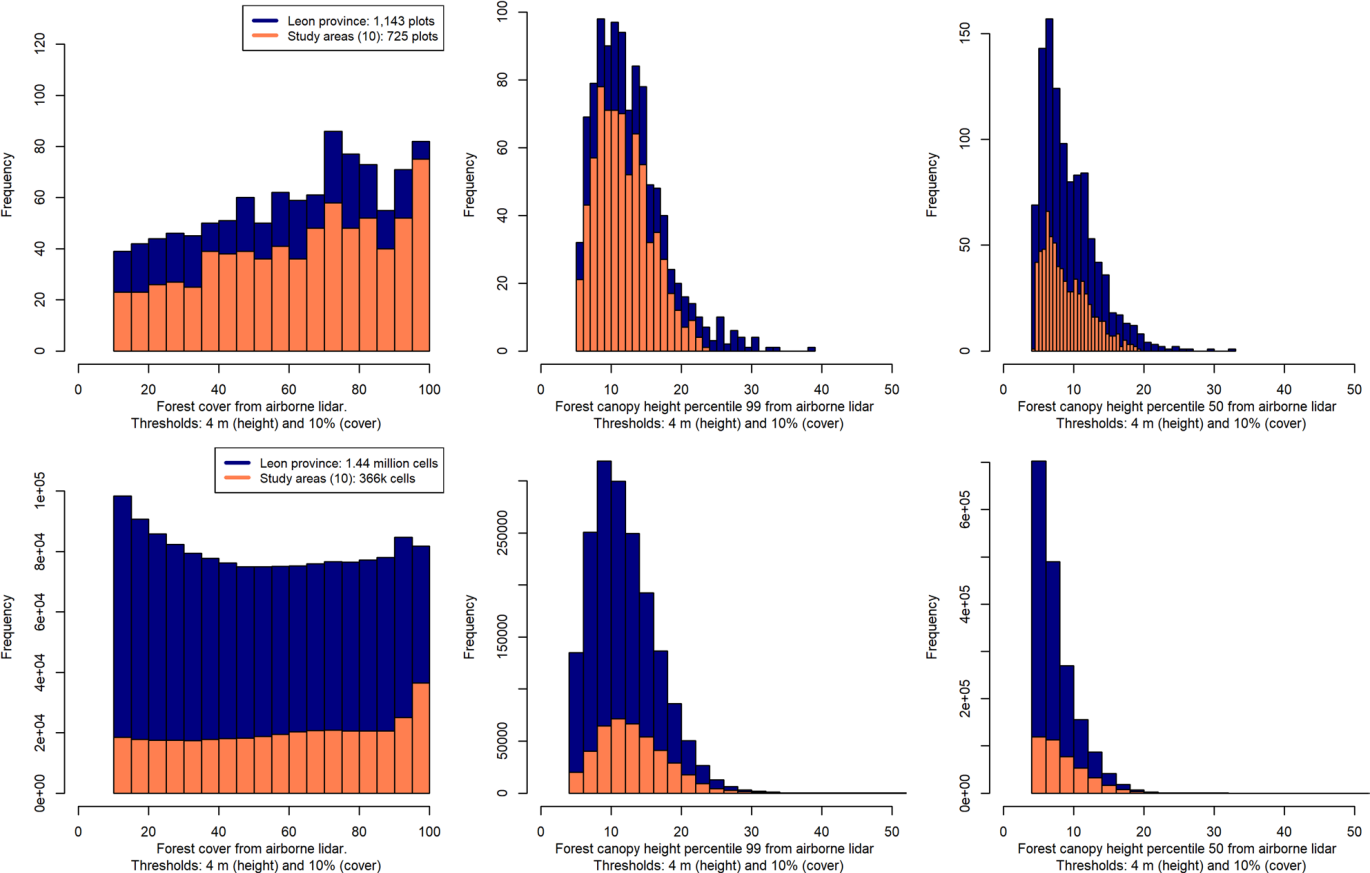
Distribution of airborne LiDAR metrics on height and cover similarly computed for NFI plots (above) and for a 44-m grid equivalent to the size of NFI plots (25-m radius) used to summarize wall-to-wall LiDAR. Results are presented for Leon province (jurisdiction) and the 10 most-sampled municipalities (sub-jurisdictions)

In the previous estimation phase, the AGBD-ALS based models were calibrated using 25-m radius NFI inventory plots, equivalent to a square grid cell of 44-m side. The entire Leon province was ALS-gridded at the scale of NFI plots before testing another four canopy height products: three ALS grids of 10, 25 and 100 m generated by the authors using the filters on height and cover described previously (Sect. "Material and methods") and one external product: the 30-m biomass product from Planet [41]. The scales of 10 and 100 m match the Lang’s global CHM and the CCI-Biomass product [62], respectively. The multi-resolution grids of ALS metrics on canopy height and cover helped to evaluate the sampling range covered by the NFI, showing limitations to include low-stature and low-forest cover areas as samples (Fig. 2) – we discuss later its impact from the optics of ALS-aided calibrations.

**Fig. 3:**
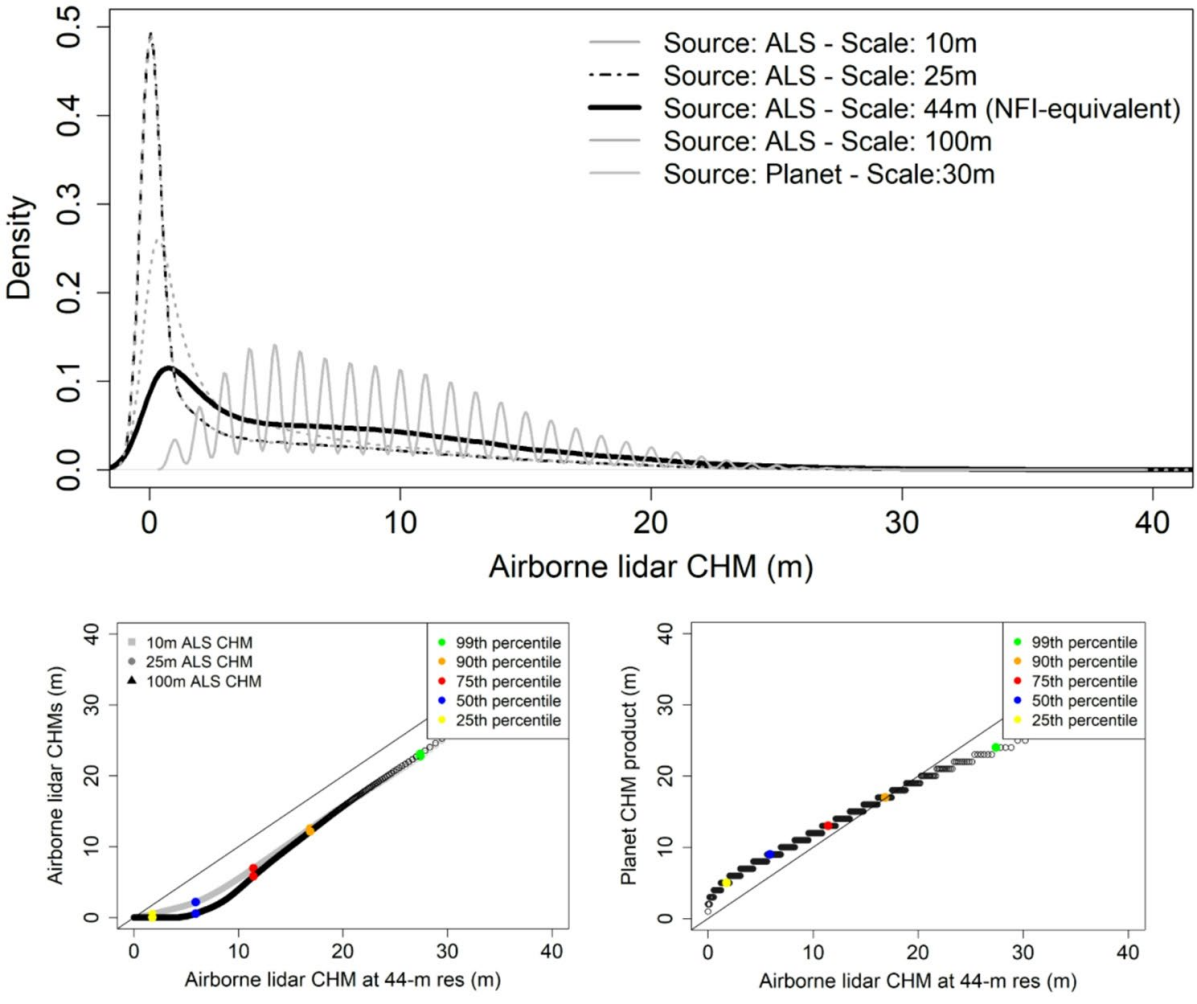
Distribution of canopy height estimates from airborne LiDAR metrics for a jurisdictional area in Spain. Results are presented for LiDAR grid estimates and the commercial Planet product (height estimates are provided as integer values) using as baseline the ALS grid of 44 m that is area-equivalent to the size of NFI plots used for biomass calibration

The generated CHMs for the ALS grids and the Planet product were compared in terms of quantiles distribution to depict systematic biases in the products. We used the ALS grid of 44 m as baseline for comparing ALS grids that violate the area-equivalency of NFI plots and also the commercial Planet map generated as a non-continuous integer product that overpredicts forest height for the low range (< 10 m) and underpredicts for mature stands (Fig. 3). ALS-Biomass based models were applied across Leon province. The assignment of the stratums-specific biomass models to each ALS grid cell was based on the Spanish Forest Map (SFM) that assigns stratum information to NFI samples and all forests areas in Spain. Stratum-level biomass predictions were aggregated to produce on jurisdictional biomass map for the four resolutions explored.

### Methods to estimate mean and total biomass at sub-jurisdictional level

We compared three methods to infer mean biomass at two hierarchical planning levels: (i) sub-jurisdictions (municipalities) and (ii) forest strata boundaries. The first boundaries were administrative while the second were the strata used by the NFI for official reporting and were retrieved from the SFM. The distribution of NFI plots is presented in Table S2. We selected the 10 municipal boundaries – similar number used in [54] - with the highest number of NFI plots (>20) in the province.

The first method tested was the traditional design-based estimation using only National Forest Inventory (NFI) plot data to calculate mean and total biomass stocks. The second method applied a model-based (MB) approach, using biomass calibration models derived from NFI plots and airborne laser scanning (ALS) metrics at regional level to produce wall-to-wall biomass predictions across the study areas. As a third alternative, we implemented small-area estimation (SAE) techniques specifically designed to generate robust estimates and confidence intervals in domains with limited ground-truth observations. The following sections provide a detailed description of each approach.

#### Estimation of design-based method using NFI plots (DB)

The design-based estimator of the stratum-specific biomass mean was the sample mean for the plots within the stratum:

1 $$\:{\widehat{\stackrel{-}{Y}}}_{DB}=\frac{\sum\:_{i=1}^{n}{y}_{i}}{n}$$

where $\:{\mathrm{y}}_{\mathrm{i}}$ is the field biomass for the plot *i*, and *n* is the number of plots for each stratum. The NFI sample is obtained using a systematic sampling design, thus, to estimate the variance of $\:\stackrel{-}{Y}$ we followed [67] and resorted to formulas for simple random sampling:

2 $$\:\widehat{Var}\left({\widehat{\stackrel{-}{Y}}}_{DB}\right)=\frac{{s}_{y}^{2}}{n}$$

where

3 $$\:{s}_{y}^{2}=\frac{\sum\:_{i=1}^{n}{\left({y}_{i}-{\widehat{\stackrel{-}{Y}}}_{DB}\right)}^{2}}{n-1}$$

The corresponding estimator of the SE was computed as follows:

4 $$\:{\widehat{SE}}_{{\widehat{\stackrel{-}{Y}}}_{DB}}=\sqrt{\widehat{Var}\left({\widehat{\stackrel{-}{Y}}}_{DB}\right)}$$

Absolute and relative errors are computed as indicated in Eqs. 5 and 6.

5 $$\:\epsilon\:=t\cdot\:{\widehat{SE}\:}_{{\widehat{\stackrel{-}{Y}}}_{DB}}$$

6 $$\:{\widehat{SE}\:}_{{\widehat{\stackrel{-}{Y}}}_{DB}}\:\left(\%\right)=\frac{\epsilon\:}{{\widehat{\stackrel{-}{Y}}}_{DB}}\cdot\:100$$

Although the estimators shown above are not-unbiased when applied to systematic samples ([68], p. 55), they are widely used and are conservative in the sense of estimating a greater variance for the population mean [69].

#### Population-level model-based Estimation (MB)

Following McRoberts, (2006) we applied the standard model based estimator, i.e., the mean of the biomass predictions for all ALS grid units (see Table 2):

7 $$\:{\widehat{\stackrel{-}{Y}}}_{MB}=\frac{1}{N}\:\sum_{i=1}^{N}{\widehat{\mu\:}}_{i}$$

where $\:{\widehat{\stackrel{-}{Y}}}_{MB}\:$is the predicted biomass value using the ALS-based potential model (Table 2) for the *i*^th^ population unit (pixel, map unit), and N is the total number of population units for each forest strata for which ALS-based biomass models were applied. The standard error (SE, Mg ha^− 1^) estimator was defined as follows:

8 $$\:{\widehat{SE}\:}_{{\widehat{\stackrel{-}{Y}}}_{MB}}=\sqrt{\frac{1}{{N}^{2}}\sum_{i=1}^{N}\sum_{j=1}^{N}{Z}_{i}^{T}{\widehat{\sigma\:}}^{2}\left\{\widehat{\beta\:}\right\}{Z}_{j}}$$

where $\:{z}_{ij}$= $\:\partial\:f\left({X}_{i},\beta\:\right)/\partial\:{\beta\:}_{j}$ are the elements of vector $\:{Z}_{i}$, $\:{\beta\:}_{j}$ are the parameters of the model (Eq. 1), and $\:{\widehat{\sigma\:}}^{2}\left\{\widehat{\beta\:}\right\}$ is an estimator of the parameter variance-covariance matrix as described in McRoberts, Næsset, and Gobakken (2013).The elements $\:{z}_{ij}$are the partial derivative of the model in position *i* (number of parameter) regarding the *j* parameter. The function and routines to calculate the SE were implemented in R software [72] using the raster-based metrics included into the ALS-based models. In addition, absolute and relative errors were computed as follows:

9 $$\:\epsilon\:=t\cdot\:{\widehat{SE}\:}_{{\widehat{\stackrel{-}{Y}}}_{MB}}$$

10 $$\:{\widehat{SE}\:}_{{\widehat{\stackrel{-}{Y}}}_{MB}}\:\left(\%\right)=\frac{\epsilon\:}{{\widehat{\stackrel{-}{Y}}}_{MB}}\cdot\:100$$

where *t* is the critical value of the t-student distribution with *N*−1 degrees of freedom for a 95% confidence level. We also calculated for each of the estimators the empirical coverage of the confidence interval (*CI*). That is, for each domain or strata, we counted the proportion of samples where the true mean was included in the estimated *CI*:

11 $$CI=\:{\widehat{\stackrel{-}{Y}}}_{MB}\pm t\cdot{\widehat{SE}\:}_{{\widehat{\stackrel{-}{Y}}}_{MB}}$$

#### Estimation for small areas using NFI plots and ALS grids (SAE)

The implemented small area estimation (SAE_ALS+NFI_) method does not necessarily refer to small spatial areas but rather to areas that contain little or no ground samples [73, 74]. In national forest inventories (NFIs), data is collected through a systematic or random sample design. While estimates at the national or regional level are often reliable due to larger sample sizes, smaller units may have too few or no sample plots, leading to high uncertainty at municipality level [7]. SAE addresses this issue by borrowing strength from related areas and auxiliary data sources. Implementations based on SAE and ALS-based forest inventory have a suitable niche for small jurisdictions lacking enough NFI sampling plots [57, 58, 75, 76]. However, more research is necessary for areas covered by very low NFI samples (Table S2) in order to know the limitation and precision of SAE method comparing with an external MB calibration fitted with many more training plots.

The SAE approach relates ground measurements of biomass and auxiliary data (ALS metrics). We matched the scales in the crossovers between ALS grid metrics at 44 m and ground plots (25-m radius). The two-phase implementation of SAE available in the ‘forestinventory’ package developed for the R environment [74] was executed for this third alternative.

The first phase is associated with auxiliary ALS information used to generate model predictions based on a linear regression (ordinary least squares, OLS). The second phase contains the NFI plots for biomass calibration, that was used to generate model coefficients and correct bias. Aligned with the variable selection method used in plot-level biomass calibrations, we selected metrics for height: top-of-canopy (*h*_*99*_) and average height (*h*_*mean*_); and forest cover (*FC*).

The unit-level SAE method was performed using the two-phase exhaustive method described in [74] scoped for two calculation units: individual strata within each municipality and single aggregation by stratum and by municipality.

Following [74], we used the following equations to estimated mean biomass (Eq. 12), its variance (g. variance, Eq. 12) and the SE (Eq. 14) for inter-comparisons of uncertainty:

12 $$\:{\widehat{\stackrel{-}{Y}}}_{G,SAE}={\stackrel{-}{X}}_{G}^{T}{\widehat{\beta\:}}_{s2}+\frac{\widehat{R\:}}{{n}_{2,G}}$$

13 $$\begin{aligned} &\:\widehat{Var}\left({\widehat{Y}}_{G,SAE}\right)={\stackrel{-}{X}}_{G}^{T}{\widehat{\sum\:}}_{\widehat{\beta\:}}\:{\stackrel{-}{X}}_{G} \\&\quad+{\stackrel{-}{\beta\:}}_{S2}^{T}{\widehat{\sum\:}}_{\widehat{{\stackrel{-}{X}}_{G}}}\:{\widehat{\beta\:}}_{s2}+\frac{1}{{n}_{2,G}}{\widehat{Var}}_{s2,G}\left(\widehat{R\:}\right)\end{aligned}$$

14 $$\:{\widehat{SE}}_{{\widehat{Y}}_{G,SAE}}=\sqrt{\widehat{Var}\left({\widehat{Y}}_{G,SAE}\right)}$$

where the predictions over the small area G (Eq. (12), first term) are corrected by the mean bias of the model$\:\frac{\widehat{R\:}}{{n}_{2,G}}$. The variance of the predictions $\:{\widehat{\stackrel{-}{Y}}}_{G,SAE}$ is estimated by the sum of three terms, the first one is related to the variance–covariance matrix ^$\:{\widehat{\sum\:}}_{\widehat{\beta\:}}$of the regression coefficient $\:{\widehat{\beta\:}}_{s2}$, estimated on *s*_*2*_, the second term to the variance–covariance matrix ^$\:{\widehat{\sum\:}}_{\widehat{{\stackrel{-}{X}}_{G}}}$of the auxiliary vector $\:{\stackrel{-}{X}}_{G}$, and the last term to variance of the residual correction term$\:\:\frac{\widehat{R\:}}{{n}_{2,G}}$. Mean biomass estimates and SE uncertainty values were computed for the two calculation units at stratum-level. The absolute and relative error were computed as in Eq. 5 and Eq. 6.

### Upscaling of biomass mean estimates to total stocks

The stratum-average biomass predictions using ALS grids and NFI plots were upscaled to total values for sub-jurisdictions (Table S1) for inter-comparisons of biomass statistics used in accounting and reporting [9, 11]. For design-based NFI approach and the SAE implementation, we multiplied mean biomass estimates over the extent of each forest strata as in the official SFM. However, for MB estimation using multi-resolution ALS grids, we estimated the biomass weight of each prediction before summing totals within strata boundaries – this approach maximizes the ability of ALS data to discriminate and account for (i) low-stature vegetation such as grasslands or shrubs not sampled by the NFI, (ii) presence of gaps within the extent of forest patches, (iii) filter out laser pulses not classified as forest vegetation that can still be present within forest type boundaries and thematic forest maps in general.

## Results

### Comparisons of approaches for mean biomass Estimation

**Fig. 4:**
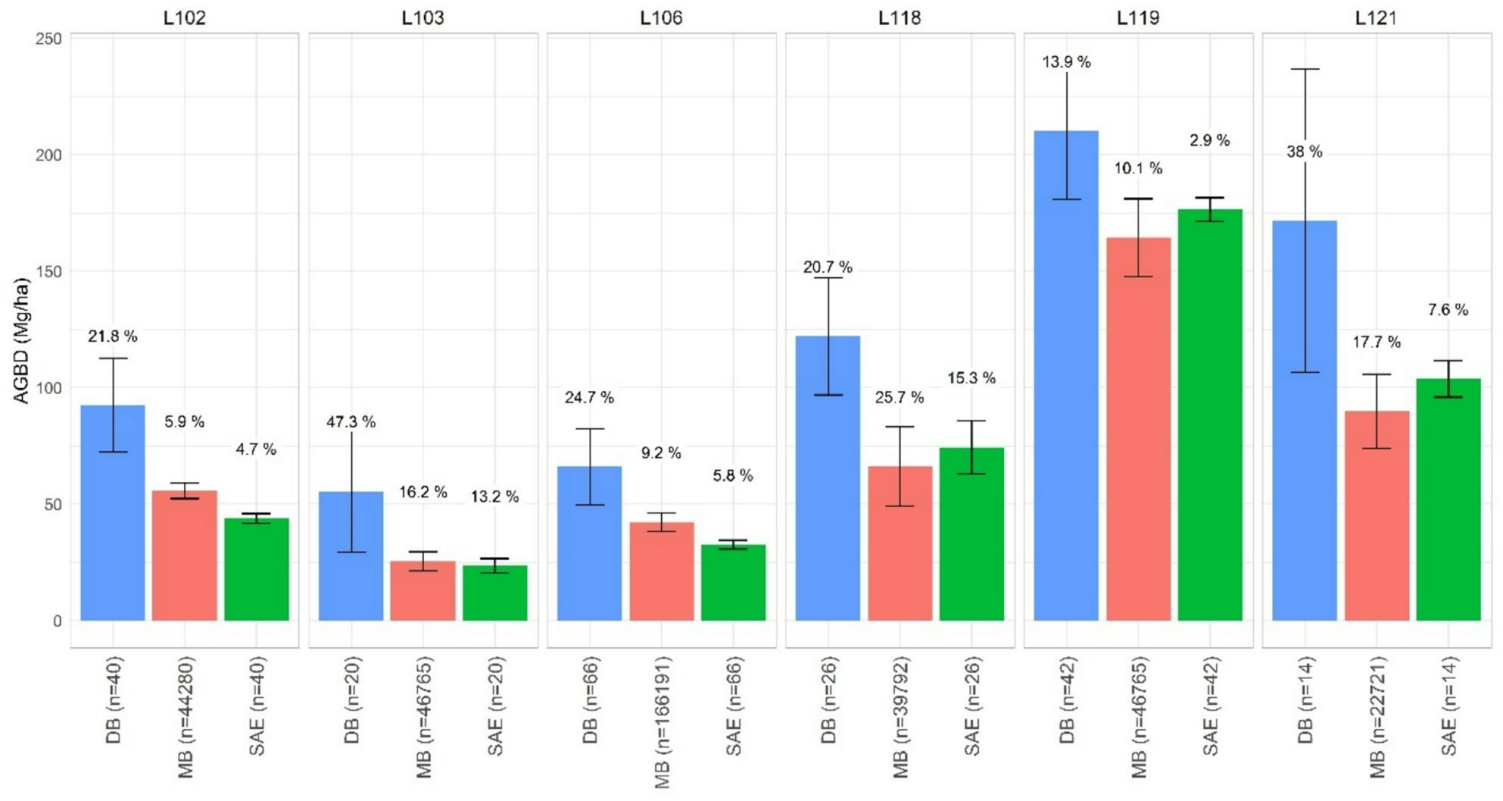
Mean biomass estimates (AGBD, Mg ha^− 1^) and their relative standard error (SE, %) for NFI design-based (DB), model-based (MB) estimation and small-area estimation (SAE). Results are presented for strata aggregations across sub-jurisdictions. Values represent the SE (%) and bars define the confidence interval (95% probability with Student’s *t* distribution = 1.96) calculated as mean biomass $\:\pm\:\:t\cdot\:{\widehat{SE}\:}_{\widehat{\mu\:}}.)\:$

The performance of the DB estimator was not aligned to estimates from MB and SAE (Fig. 4). Overall, estimates in the DB approach were notoriously higher compared to the two ALS-aided options. Between these two and in terms of uncertainty, the MB method showed slightly higher values than SAE for all formations. However, uncertainty in SAE inference was substantially lower than MB for some cases, specifically for *P. pinaster* (L103), *P. nigra* (L118) and *Quercus* spp (L121) strata for which the available NFI sampling was scarce and sub-optimal for robust MB inference.

**Fig. 5:**
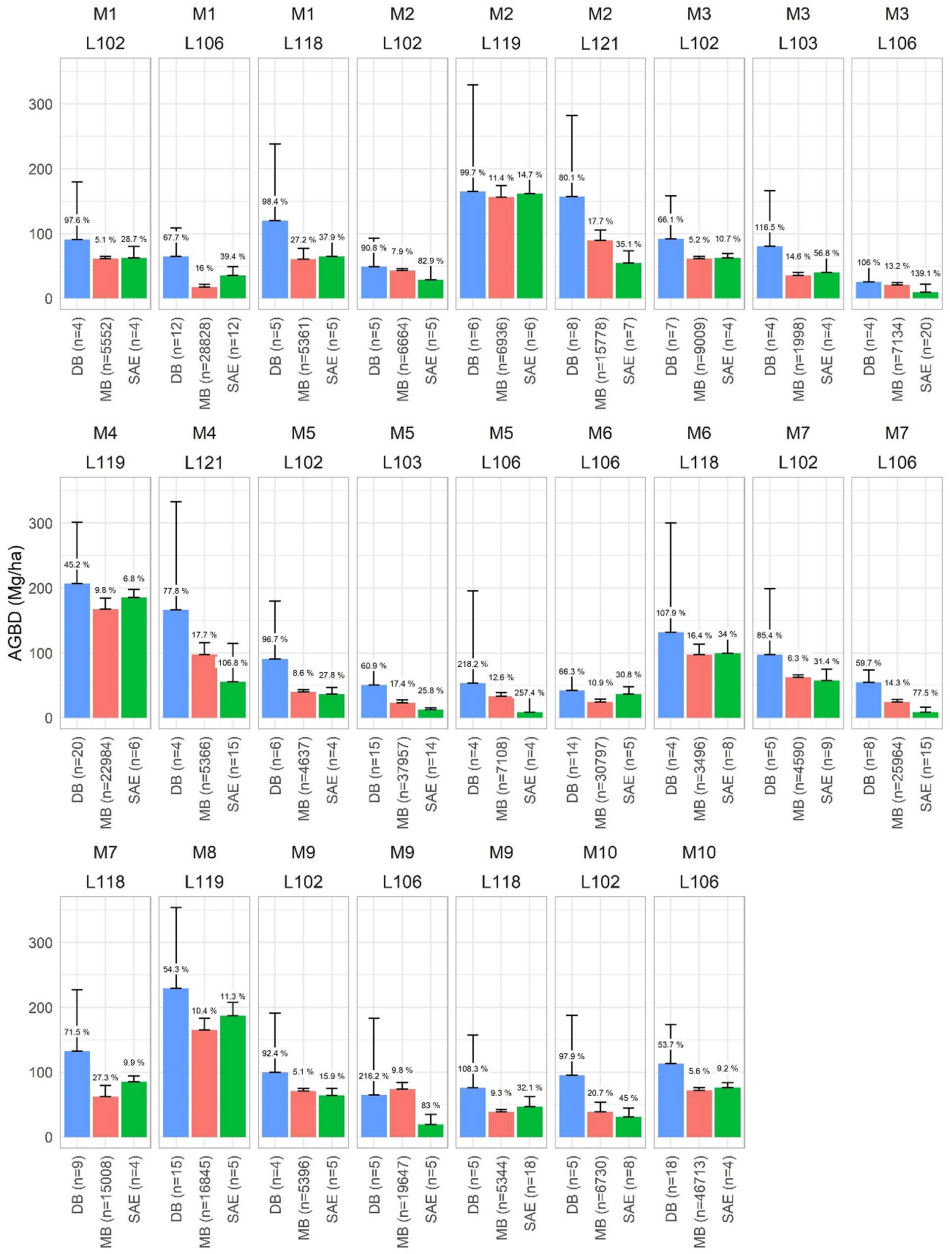
Mean biomass estimates (AGBD, Mg ha^− 1^) and their relative standard error (SE, %) for NFI design-based (DB), model-based (MB) estimation and small-area estimation (SAE). Results are presented at stratum-level within each sub-jurisdiction evaluated. Values represent the SE (%) and bars define the confidence interval (CI, 95% probability with Student’s *t* distribution = 1.96) – only the non-negative side of the CI is presented

The downscaling of estimates at the individual stratum – between 2 and 6 stratum boundaries per municipality – decreased even more the precision of estimates from DB (Fig. 5). For instance, estimates from SAE exceeded the estimated mean biomass (SE > 100%) and we found these estimates were more uncertain at this scale compared to MB inference despite the former calibrates biomass using local NFI data rather than using a model trained with very few local but mostly with NFI plots not included in the sub-jurisdictional domains evaluated. The uncertainty in SAE estimates significantly increased for cases containing less than five NFI plots.

### Scaling effects in biomass predictions

**Fig. 6:**
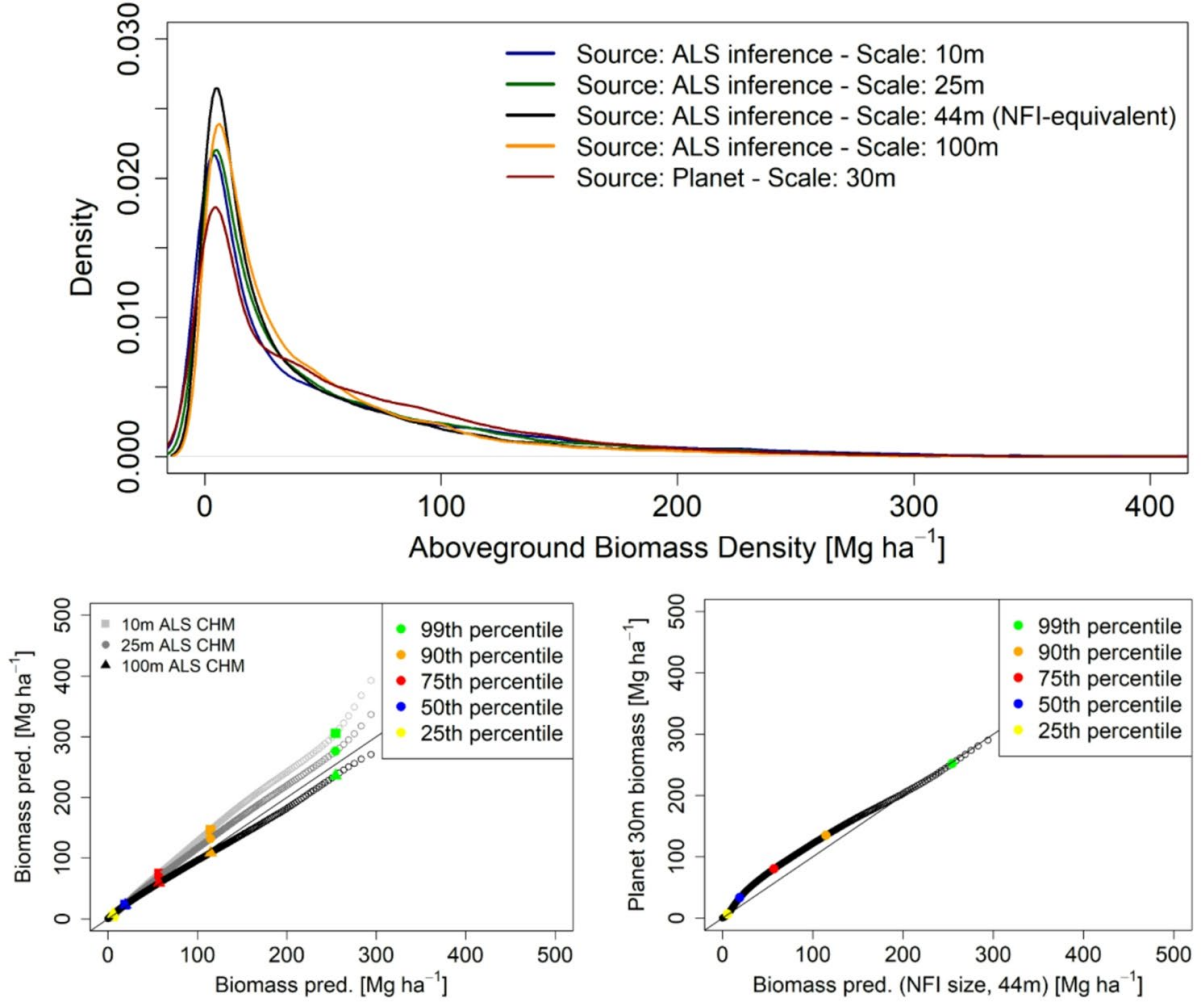
Distribution of aboveground biomass density (AGBD, Mg ha^− 1^) predictions from calibration models applied over grids of airborne laser scanning (ALS) metrics. Inter-comparisons of ALS-based inferences uses the grid scale of 44 m (equivalent to NFI plot sampling size) as baseline. We include the Planet biomass map developed for Europe at 30-m scale in the experiment

The domain of biomass predictions using MB showed a strong overall alignment but distributions over the baseline grid of 44 m stressed the impact of scaling: biomass estimates were significantly higher for 10-m biomass predictions compared coarser scales. Similar distributions were observed in the ~ 25 m range between MB inference the commercial Planet product, both aligned well to our baseline. The Planet CHM map treats biomass as a continuous variable and not as an integer (Fig. 6) - an improvement in biomass prediction added to a successful mitigation of two issues in the CHM product: saturation and over-prediction of short-stature vegetation.

**Fig. 7:**
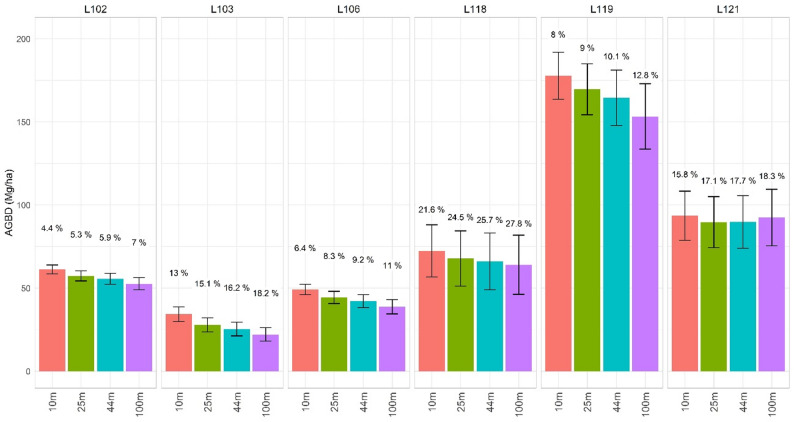
Mean biomass estimates (AGBD, Mg ha^− 1^) and uncertainties by means of relative standard error (SE, %) on the mean for MB-based inference at different prediction scales. Values represent the SE (%) and the bars define the confidence interval (95% probability with Student’s *t* distribution = 1.96) calculated as mean biomass $\:\pm\:\:t\cdot\:{\widehat{SE}\:}_{\widehat{\mu\:}}.)\:$– both sides of the CI are presented

Scale-varying estimates for MB inference were high for cases lacking enough representation of NFI plots i.e., *P. pinaster* (L103), *P. nigra* (L118) or *Quercus spp* (L121). We found an increasing pattern for the relative SE with respect to the scale of the support area used to calculate ALS predictors (Fig. 7): The difference in term of relative SE ranged between 2.6% and 6.2% across both the strata and scales, from the finest 10 m to the CCI-scaled 100 m. Our results indicated that the coarser the scale of biomass predictions the more pronounced the underestimation is with respect to the baseline (model-based (MB) estimation at 44-m scale to match the resolution of NFI sampling plots. The opposite tendency e.g., overestimation as scale becomes finest, was confirmed. These patterns were consistent across the six strata assessed.

### Total biomass for jurisdictional reporting

**Fig. 8:**
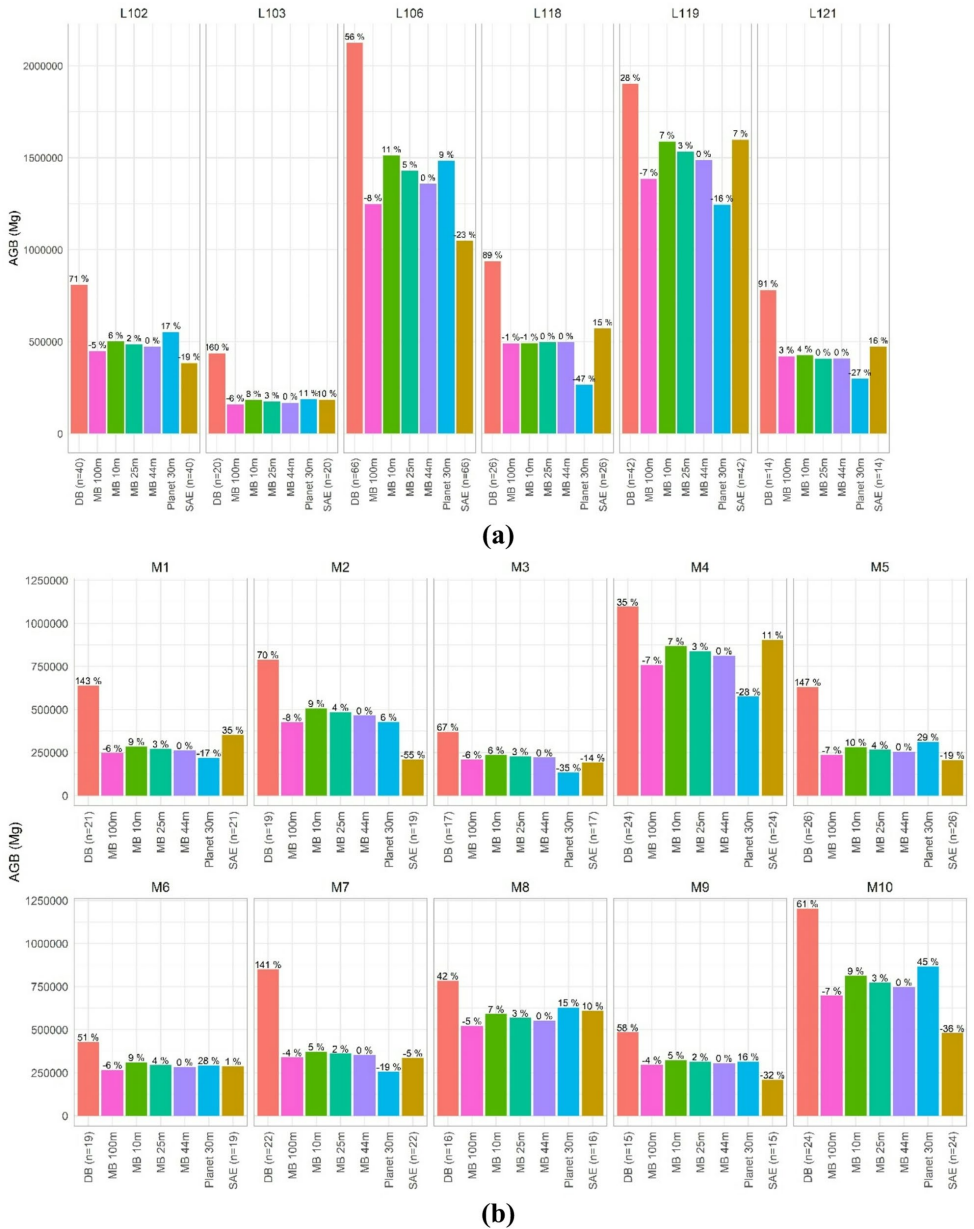
(**a**) Total biomass (Mg) estimated for strata (above) and (**b**) sub-jurisdictions (below). Estimates from (i) NFI- design based (DB), (ii) small-area estimation (SAE), (iii) four examples of model-based (MB) estimation at different resolutions and (iv) the commercial biomass product from Planet in Europe, are compared. Above-bar values express the relative change of the estimate over the baseline case (MB at 44-m) that matches the size of NFI plots. The number of NFI plots used for each estimate are also presented

Following the results on mean biomass estimation, total stocks estimates were highly uncertain for DB compared to predictions aided with ALS, either for MB or SAE. In terms of strata, the maximum deviation computed for MB was 11% of the relative mean bias at the 10-m scale in stratum L106 (Fig. 8a). Differences in total biomass range from positive 5% for the case of 25-m predictions to negative 8% for 100-m predictions over the baseline (Fig. 8a). Factors such as predictions scaling or the performance of the SAE did not follow a systematic pattern when results were aggregated for the six strata. For the SAE approach total biomass significantly oscillated from − 55% to 35% around the baseline for both stratum (Fig. 8a) and sub-jurisdictional summaries (Fig. 8b). In some cases, biomass totals were nearly invariant towards different prediction scales (stratum L119) but in other cases the effect was pronounced as observed for jurisdictional summaries. Totals from DB doubled the estimates from MB inference regardless of predictions scale in 5 of the 10 sub-jurisdictions assessed. Tendencies were similar between MB means and totals – the later estimated by summing the biomass weight of individual pixels.

## Discussion

### Scaling dependencies in biomass Estimation and prediction

Switches in scale of canopy height metrics (25 m and 100 m) from ALS had a low-moderate impact on biomass predictions and on the main statistics used for jurisdictional biomass reporting: mean and total stocks. The impact was greater for the finest scale (10 m) compared coarser choices. Our baseline changes for forest strata ranged from negative − 8% to positive 5% when using 100 m and 25 m as prediction scales, respectively (Fig. 8). Our values are higher compared to [46] who reported a maximum bias of 3% for a more local experiment - scales between 8 m and 25 m – based on size-varying sampling plots. The maximum relative bias reached 11% for 10-m predictions compared to the NFI-native resolution of 44 m. Our maximum value is high but slightly lower than the 15% bias reported in [37] using pixel sizes between 5 m and 70 m. Hence, the resolution effect must be meticulously accounted for in ALS-assisted large-scale inventories conceptualized to provide unbiased estimations.

Our results for the MB_*ALS+NFI*_ alternative (Fig. 7) showed that large sample sizes decrease errors when fitting ALS-based calibration models [7, 77]. Our estimates of uncertainty help to understand the consequences of predicting biomass using models calibrated at the scale of the NFI sampling. This is a relevant matter considering the increasing availability of canopy height products – and flexibility of methods to create those – from which to predict biomass using relations between canopy height profiles and ground-based biomass data [51]. A thoughtful assessment of the NFI sampling properties that affect biomass calibrations and predictions is recommended before digging into comparisons of mean and total estimates.

### Uncertainty in NFI sampling for sub-jurisdictional boundaries

The precision and performance of NFI samplings must be evaluated at appropriate scales. In Spain, the NFI is designed for 1-km grid countrywide. At the national or regional scale, sampling coverage is enough for trustworthy estimates of forest metrics such as biomass [7]. However, for lower jurisdictional hierarchies such as municipalities the sampling is not capable to provide reliable estimates with consistency. Our report on high uncertainties for mean and total biomass are especially insightful for managers and stewards aiming to assess available forest carbon biomass with inventory data. Specifically, the uncertainty in DB was above 20% in all cases except for a single stratum (L119, Fig. 4). The magnitude of these SE values exceeded mean estimated values in several cases, mostly where NFI coverage is low. How low the NFI coverage should be to discard estimates? The question is of the highest interest for forest carbon monitoring systems aiming to provide reliable estimates for more operational planning scales [4, 78, 79]. Our evidences reinforce the idea that adding auxiliary information alleviates data constraints that are evident when using only NFI samples to estimate biomass [36, 53, 57–61]. The NFI data coverage within jurisdictions has a major influence on inter-comparisons of biomass statistics and methods, but other intrinsic factors of the NFI sampling were also found to have an impact on biomass estimation and reporting statistics.

The sampled gradient of forest canopy height sensed by airborne ALS in the NFI was satisfactory for Leon province, but the NFI-sampled locations present a slight skewedness towards dense forest conditions. In previous studies, authors and many others, have favored the selection of both height and coverage metrics in calibration models assuming the unbiased on the NFI sampling [7, 23]. Both groups of variables are usually explored as predictor variables for calibrations of area-level forest variables [30, 31]. Variable selection is relevant for the purpose of explaining differences among approaches i.e., between DB and MB. Forest low-density conditions (FC < 30%) were less represented in the training data. The full coverage of detailed ALS point clouds allows to sense sparse low-density vegetation and short-stature vegetation across the landscape. Over these conditions we applied models usually calibrated with forest cover metrics from ALS data. We could have modified the composition of the biomass models by narrowing predictor selection to ALS height percentiles metrics at the cost of losing FC predictive power and increasing the error. Our choice of keeping the models as in previous studies aims to raise a flag on this particular issue that justifies the integration of ALS metrics in field sampling and pre-sampling stages. As of today, the NFI assesses aerial images contemporary to field measurements to decide whether a given plot ranges over a forest area (FC >10% following the FAO definition of forest area). Aerial imagery interpretation is still used to define FC in the plots. Discrepancies between FC obtained by photointerpretation and from ALS metrics – defined as the proportion of laser pulses over a given height-break – can be addressed by integrating the more-accurate ALS cover metrics as attributes in the field plots.

### Benchmarking ALS-supported estimates

High-density ALS point clouds capture variability, edges and gaps that impact the averaging of canopy height estimates or biomass predictions for a given boundary. It is normal to expect lower mean biomass for approaches such as the MB relying on ALS data and filtering algorithms to discriminate forests from other vegetation [80] compared to a sampling design that tends to allocate plots over dense structural conditions. Our results at this scale demonstrated that SAE performed better than MB inference that relies on more “external” plots not ranging with the assessed domains: estimates for aggregated strata across sub-jurisdictions were better for SAE compared MB and DB (Fig. 4). The SAE arena powered with ALS data information is highly valuable for small operational scales such as medium-large management units. Similar results were reported in other studies based on SAE and NFI data in i.e., Norway [57, 58, 75, 76] or the United States [70–82]. However, when we tested SAE at the lowest scale (i.e., individual strata within each municipality) these were better than the DB estimator but worse than MB (Fig. 5). This indicates that for areas covered by very low NFI samples, the mean estimator in SAE is less precise than using an external MB calibration fitted with many more training plots.

The robustness of MB models is an advantage over local conditions when the sampling driving the MB inference is representative enough of the population [54]. Consequently, the definition of a ‘small area’ requiring estimates is context-dependent, influenced by both the overall size of the study area and the available sampling fraction. Despite MB performing better than SAE at the finest domains, the uncertainty from SAE was below ~ 15% when using the NFI-native resolution to summarize ALS metrics (44 m). In traditional DB, however, only one stratum showed an error below 15% (Fig. 6), which is a common threshold of admissible errors in regional forest management planning guidelines. Traditionally, samplings for forest plans must be conducted at maximum 15% in SE to get the approval for operational management and meet forest legislation in Spain. Further investigation is necessary to determinate the optimal scale size for which SAE may be preferred over MB inference. Under this angle, the SAE method was found to be more optimal for aggregated strata covering areas between ~ 4600 ha to ~ 30,000 ha under a NFI-sample fraction of 0.08%.

## Conclusions

In this article, we sought to respond first to the issue of scale invariance in biomass estimates using MB inference in the context of canopy height mapping. Impacts on the distribution and statistics of biomass estimates are tangible and expected as scales deviates from sampling sizes used in field inventories to produce calibrations. The use of LiDAR-aided methods such as MB or SAE seems the logical roadmap for NFI programs and agencies to cope with the sparsity of nationwide sampling designs to provide baseline carbon biomass estimates for sub-jurisdictions below national and regional domains. Uncertainty estimates from SAE were lower than in MB for aggregated strata while the opposite trend was observed for individual stratum for which MB inference built with a larger population shrink the error range that SAE provides for the case of boundaries containing very few field plots. The balance between sampling intensity and the desired scale to compute biomass should be considered when preferring between MB and SAE to improve the highly uncertain in DB that relies on sparse and scarce ground-truth data.

## Supplementary Information

Supplementary material 1.

## Data Availability

No datasets were generated or analysed during the current study.

## Abbreviations

CHM: Canopy Height Model
AGBD: Aboveground Biomass Density
ALS: Airborne Laser Scanning
GEDI: Global Ecosystem Dynamics Investigation
NFI: National Forest Inventory
PNOA: Plan Nacional de Orthophotography Aerea
RMSE: Root Mean Square Error
NFI-4: Spanish National Forest Inventory 4^th^ round of measurements
SD: Standard Deviation
