# Supplementary material for: Scaling and sampling dependencies of forest canopy height mapping towards jurisdictional biomass reporting using airborne LiDAR and small-area estimation
Source: Carbon Balance Manag. 2025 Dec 8;21:12. doi: 10.1186/s13021-025-00370-9 (PMC12797554; doi:10.1186/s13021-025-00370-9)
Supplement: Supplementary file 1 — Supplementary material 1. [file 13021_2025_370_MOESM1_ESM.docx]

# Supplementary

**Canopy height map from ALS for Leon Province**


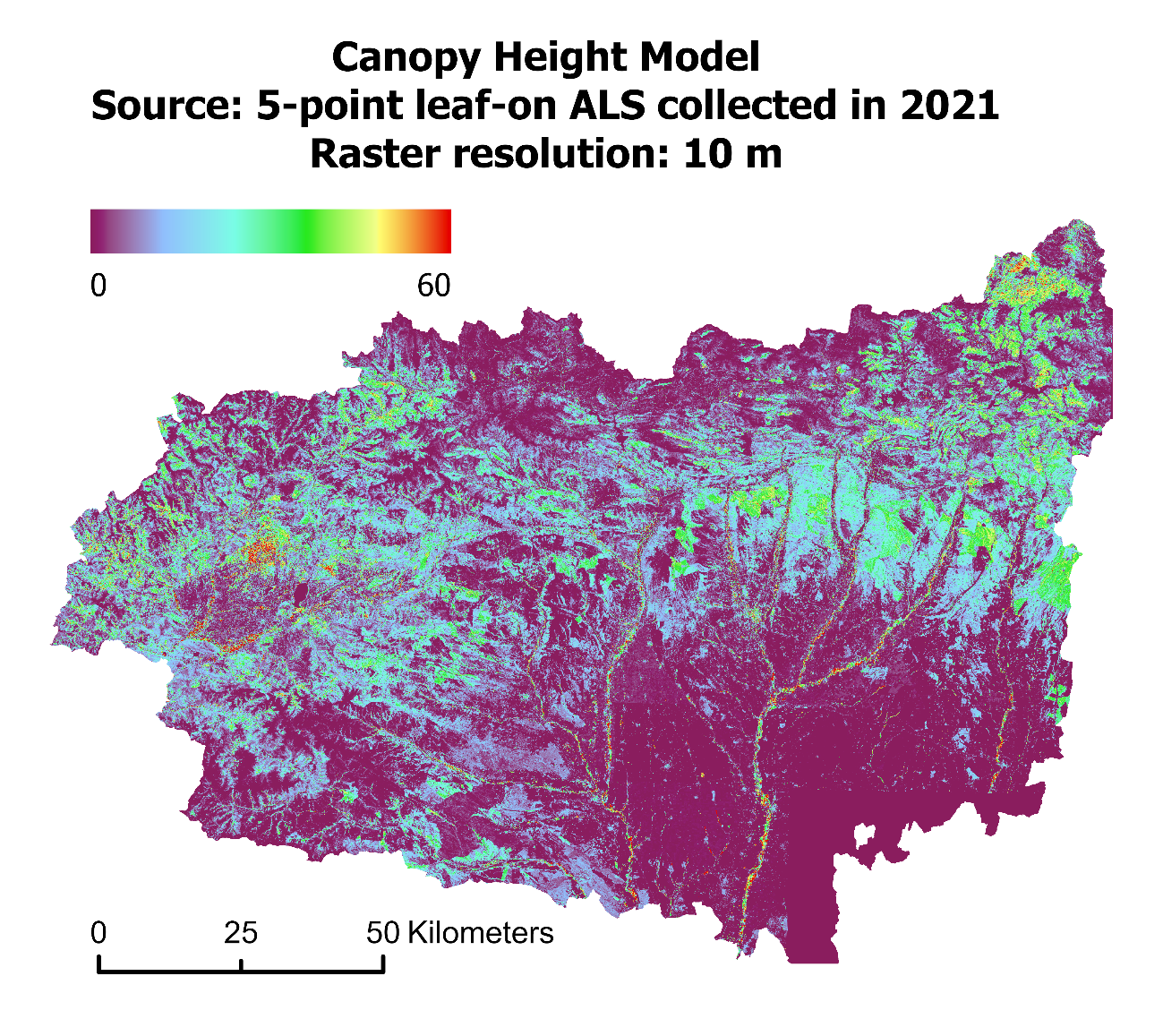


**Figure S1.** Canopy height model (CHM) derived from airborne laser scanning (ALS) data for Leon province in NW Spain. The presented resolution of the CHM product is 10 m.

**Description of sub-jurisdictional municipal domains**

**Table S1.** List of the 10 sub-jurisdictional municipalities used to compare mean and total biomass estimates from the three different approaches tested in the research**.**

| **Sub-juridictions**  **Code** | **Municipality**  **Name** | | **Area (ha)** | | **NFI plots** | |
| --- | --- | --- | --- | --- | --- | --- |
| M1 | Almanza | | 7695.51 | | 21 | |
| M2 | Boca de Huergano | | 6029.01 | | 19 | |
| M3 | Brazuelo | | 5038.59 | | 17 | |
| M4 | Buron | | 5485.76 | | 24 | |
| M5 | Castrocontrigo | | 9888.40 | | 26 | |
| M6 | Garrafe de Torio | | 6958.76 | | 19 | |
| M7 | Gradefes | | 8820.24 | | 22 | |
| M8 | Posada de Valdeón | | 3568.76 | | 16 | |
| M9 | Santa Colomba de Curueño | | 6018.31 | | 15 | |
| M10 | Valderrueda | | 10592.23 | | 24 | |
|  |  |  | |  | |  |

**Table S2.** List of the 10 sub-jurisdictional municipalities by strata used to compare mean and total biomass estimates. The number of available NFI plots and the proportion of sampled area are presented for within each domain. The identification of sub-jurisdictional domains is listed in Table S1.

| **Sub-juridictions** | **Strata** | **NFI plots** | **Area (ha)** | **Sampled area (%)** |
| --- | --- | --- | --- | --- |
| M1 | L102 | 4 | 1,074.21 | 0.07 |
|  | L106 | 12 | 5,585.52 | 0.04 |
|  | L118 | 5 | 1,035.79 | 0.09 |
| M2 | L102 | 5 | 1,479.89 | 0.07 |
|  | L119 | 6 | 1,343.36 | 0.09 |
|  | L121 | 8 | 3,205.77 | 0.05 |
| M3 | L102 | 7 | 1,742.26 | 0.08 |
|  | L103 | 4 | 387.48 | 0.20 |
|  | L106 | 4 | 1,378.80 | 0.06 |
| M4 | L119 | 20 | 4,446.14 | 0.09 |
|  | L121 | 4 | 1,039.62 | 0.08 |
| M5 | L102 | 6 | 895.24 | 0.13 |
|  | L103 | 15 | 7,345.02 | 0.04 |
|  | L106 | 4 | 1,379.58 | 0.06 |
| M6 | L106 | 14 | 5,956.12 | 0.05 |
|  | L118 | 4 | 674.69 | 0.12 |
| M7 | L102 | 5 | 892.18 | 0.11 |
|  | L106 | 8 | 5,033.97 | 0.03 |
|  | L118 | 9 | 2,894.09 | 0.06 |
| M8 | L119 | 15 | 3,263.79 | 0.09 |
| M9 | L102 | 4 | 1,045.69 | 0.07 |
|  | L106 | 5 | 3,803.83 | 0.03 |
|  | L118 | 5 | 1,034.75 | 0.09 |
| M10 | L102 | 5 | 1,305.04 | 0.08 |
|  | L106 | 18 | 9,042.84 | 0.04 |
| **Combined** | **Average** | **7.84** | **2,691.43** | **0.08** |
